# Loss of *noggin1*, a classic embryonic inducer gene, in elasmobranchs

**DOI:** 10.1038/s41598-024-54435-9

**Published:** 2024-02-15

**Authors:** Galina V. Ermakova, Irina V. Meyntser, Andrey G. Zaraisky, Andrey V. Bayramov

**Affiliations:** 1 Shemyakin-Ovchinnikov Institute of Bioorganic Chemistry, Russian Academy of Sciences, Moscow, 117997 Russia; 2 Moskvarium Center for Oceanography and Marine Biology, Moscow, 129223 Russia; 3 Pirogov Russian National Research Medical University, Moscow, 117997 Russia

**Keywords:** Evolutionary developmental biology, Developmental biology, Embryogenesis, Experimental organisms, Gene expression

## Abstract

Secreted proteins of the Noggin family serve as pivotal regulators of early development and cell differentiation in all multicellular animals, including vertebrates. Noggin1 was identified first among all Noggins. Moreover, it was described as the first known embryonic inducer specifically secreted by the Spemann organizer and capable of inducing a secondary body axis when expressed ectopically. In the classical default model of neural induction, Noggin1 is presented as an antagonist of BMP signalling, playing a role as a neural inducer. Additionally, Noggin1 is involved in the dorsalization of embryonic mesoderm and later controls the differentiation of various tissues, including muscles, bones, and neural crest derivatives. Hitherto, *noggin1* was found in all studied vertebrates. Here, we report the loss of *noggin1* in elasmobranchs (sharks, rays and skates), which is a unique case among vertebrates. *noggin2* and *noggin4* retained in this group and studied in the embryos of the grey bamboo shark *Chiloscyllium griseum* revealed similarities in expression patterns and functional properties with their orthologues described in other vertebrates. The loss of *noggin1* in elasmobranchs may be associated with histological features of the formation of their unique internal cartilaginous skeleton, although additional research is required to establish functional connections between these events.

## Introduction

**Figure 1:**
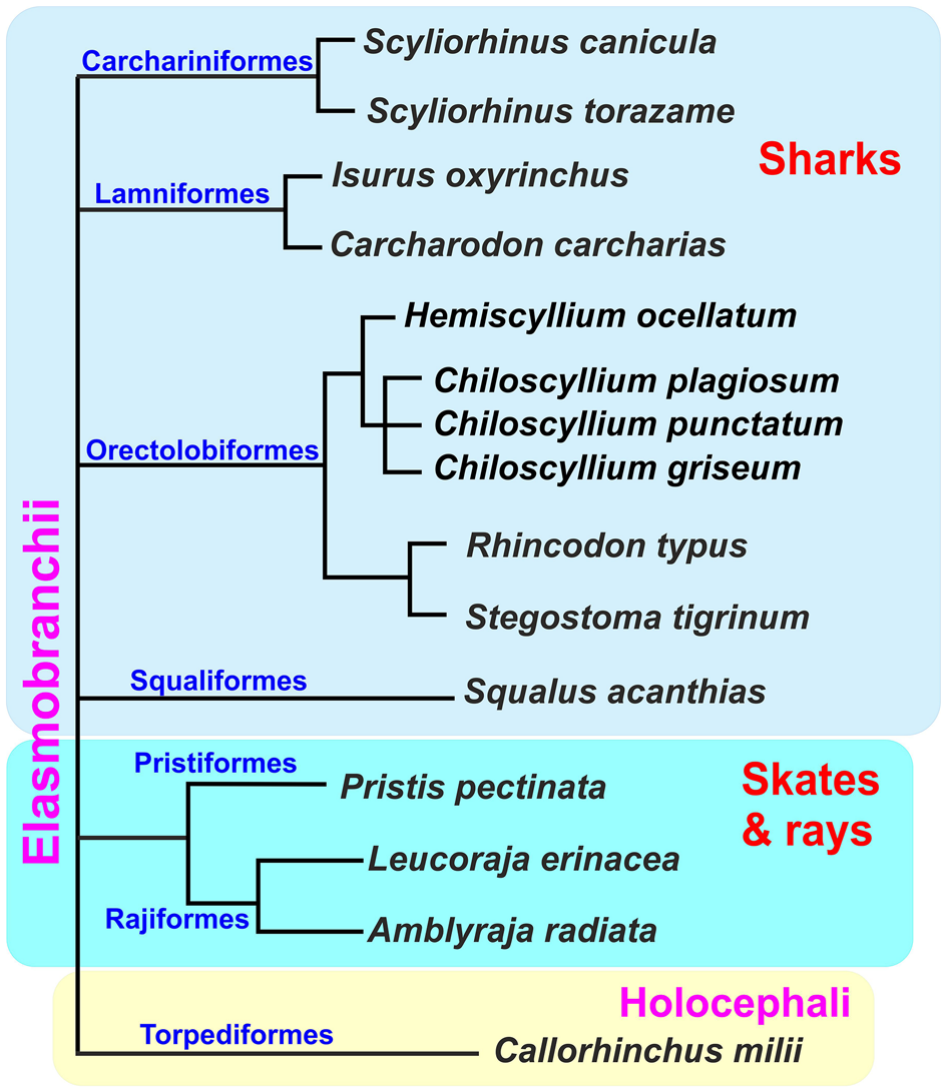
Schematic phylogeny of Chondrichthyes with the representatives, included in Noggin analysis.

Chondrichthyans are a basally divergent group of gnathostomes whose unique phylogenetic position explains the increasing research interest in them in recent years^1^. From a morphological point of view, cartilaginous fish have a number of unique features, such as the absence of a swim bladder, the presence of placoid scales homologous to the teeth of vertebrates and, of course, a cartilaginous skeleton. The chondrichthyans clade is divided into two subclasses: Elasmobranchii and Holocephali (Fig. 1). The elasmobranch group includes sharks (superorder Selachimorpha) and skates and rays (superorder Batoidea), accounting for over 1200 different species^2^. Holocephali includes chimaeras, which have fewer described species than their sister taxa, with only approximately 56 species^3^. One of the morphological differences between Elasmobranchii and Holocephali is the operculum, which is absent in the Elasmobranchii and is formed in the Holocephali by the growth of the hyoid arch rays^4^. Although the osteichthyan operculum is also derived from an outgrowth of the hyoid arch, these structures in two vertebrate lineages probably represent a case of convergent skeletal elements formed by different mechanisms—as endoskeletal appendages in chondrichthyans vs. intramembranous ossifications in osteichthyans^4^. Due to their phylogenetic position as one of the basally divergent groups of jawed vertebrates with a common ancestor that diverged from osteichthyans, approximately 450 million years ago, developmental studies of elasmobranchs have provided much insight into the process of morphological evolution of vertebrates^5,6^. Questions that are posed within the evo-devo study of sharks and rays include the origin of the jaw apparatus and paired appendages and the development of the cerebellum, dentin scales and branchial structures^6^. Laboratory studies of the early stages of shark development have a number of technical difficulties and restrictions, such as the need to keep the animals in a marine aquarium of sufficient volume, a small number of eggs, and a dense, opaque egg shell, which makes it difficult to determine developmental stages in vivo^7^. At the same time, due to their evolutionary antiquity, studies of sharks are of great value for understanding the basic mechanisms of early development of vertebrates in general.

One of the key processes in early vertebrate development is primary embryonic induction, during which patterning of the mesoderm and neuroectoderm of the embryo occurs, which leads to the formation of the rudiments of axial structures. The classical model of primary embryonic induction was developed in amphibian embryos and considers as an organizational centre a group of cells in the region of the dorsal lip of the blastopore (Spemann organizer), which is a source of signals that suppress the activity of the BMP signalling pathway. Activation of this pathway inhibits the development of dorsal structures. Chordin, Follistatin and Noggin proteins have been described as the main antagonists of BMP signalling^8–14^.

*Noggin* family genes play key roles in a wide range of developmental processes, including the development of the telencephalon, the unique part of the vertebrate brain^9,15–18^. Noggin1 was described first and has been shown to be a secreted protein capable of performing essential organizer functions. It induces the development of neural tissue in the dorsal ectoderm and dorsalizes mesoderm cells that otherwise would be involved in the composition of the ventral mesoderm^9,15,16^. Noggin1 is able to bind BMP proteins and thus inhibit the activity of the BMP signalling pathway, which is a prerequisite for the formation of neural tissue and dorsal mesoderm differentiation^13,19,20^. Due to this function, Noggin1, when expressed ectopically in the ventral part of the *Xenopus* embryo, is able to induce the formation of an additional body axis. Further research revealed that Noggin is involved in a wide range of developmental processes. Thus, the formation of dorsoventral neural tube polarity in *Xenopus* is associated with the establishment of Noggin gradients and a dose-dependent response to this gradient in explants^21^. In mice, Noggin1 synergistically with Shh participates in the induction of the homeobox gene *Pax1* during the marking of the neural tube and somitic mesoderm^22^. As a BMP repressor, Noggin1 is involved in the development of cartilage^23,24^, cells of the organ of Corti^25^, optic cup and lens^26^ and differentiation of hair follicles^27^. In experimental systems, including stem or cancer cells, Noggin1 is used as an artificial inhibitor of the BMP pathway^28^.

Although for a long time studies were devoted exclusively to the *noggin1* gene, which was considered the only *noggin* gene in vertebrates, to date, a whole family of vertebrate *Noggin* genes has been described, including four cyclostome genes (*nogginA*, *nogginB*, *nogginC*, *nogginD*) and three orthologues in gnathostomes (*noggin1*, *noggin2*, *noggin4*)^29–31^. Similarities between the genes of cyclostomes and gnathostomes have been demonstrated at the levels of amino acid sequences, genome synteny, expression patterns, and function^31^. Genes of the *Noggin* family are described in all studied vertebrates but are also present in invertebrates, playing very conserved roles. Thus, it was shown that cDNAs of the *noggin* gene of hydra (*Hydra vulgaris*) and planarians (*Schmidtea mediterranea*) can induce additional axes in the clawed frog^32,33^. Proteins of the Noggin family differ in their functional properties. Thus, Noggin1 and Noggin2 have the ability to suppress BMP and exhibit the entire spectrum of functional activities characteristic of “classical” Noggin. Moreover, the activity of Noggin1 and Noggin2 is not limited to the suppression of the BMP signalling pathway and includes the ability of Noggin to modulate the activities of the Nodal/Activin and Wnt pathways^18^. At the same time, the Noggin4 protein has a number of amino acid substitutions that impair its ability to participate in the inhibition of BMP signalling but do not prevent its binding to Wnt family ligands^34^. Because of this, Noggin4 does not have the ability, characteristic of other Noggins, to induce the formation of additional body axes, although it takes part in the formation of the head section of the embryo due to its inhibitory effect on the canonical Wnt pathway.

In this work, we analysed the presence of *noggin* genes in cartilaginous fishes and were surprised to find that the *noggin1* in this evolutionary lineage was lost in the elasmobranch clade, including sharks, skates and rays. A bioinformatics analysis using the criteria of homology and local genomic synteny did not reveal the *noggin1* in representatives of elasmobranchs. *Noggin4* has also disappeared in some elasmobranchs. At the same time, in Holocephali, which are considered a basally divergent group of the Chondrichthyes and sister group to elasmobranchs^35,36^, all three *noggin* genes characteristic of gnathostomes, including *noggin1*, were found. This indicates the secondary nature of the disappearance of *noggin1* and *noggin4* in more highly specialized representatives of elasmobranchs. In functional terms, *noggin2* of elasmobranchs exhibited properties characteristic of *noggin1/2* of gnathostomes, revealing the ability to induce the formation of additional complete body axes in *X. laevis* embryos. According to the current views, the induction of such axes, which includes the anterior cephalic structures and the simultaneous suppression of at least two signalling pathways—BMP and Wnt—is required^37,38^.

Another important result of this work is the discovery of a homologue of the *ankfn1* gene (ankyrin repeat and fibronectin type-III domain-containing protein 1) in the neighbourhood of the chondrichthyan *noggin4*. Previously, *ankfn1* was noted as a nearby gene of *noggin1/2*^31^. The presence of *ankfn1* near *noggin4* in one of the basally divergent groups of gnathostomes confirms the previous hypothesis about the origin of vertebrate *noggin*s as a result of two rounds of duplications from one ancestral gene. Previously, this scenario was considered an alternative to the scenario of two ancestral *noggins*, based on the description of *noggin-like* genes in invertebrates^33^. The main argument in favour of a single ancestral *noggin* in vertebrates was the presence of a single *noggin* in the closest relatives of vertebrates—lancelets and tunicates, and now this scenario has received additional factual confirmation.

## Results

### Phylogenetic and local genomic synteny analysis of noggin genes of elasmobranchs

We searched for homologues of *noggin* genes in Chondrichthyes in available genomic databases for *Callorhinchus milii*, as a representative of Holocephali, the sister branch to elasmobranchs, as well as for representatives of all evolutionary branches of elasmobranchs: rays (small-tooth sawfish *Pristis pectinata*), skates (thorny skate *Amblyraja radiata* and little skate *Leucoraja erinacea*) and sharks (smaller spotted catshark *Scyliorhinus canicula*, cloudy shark *Scyliorhinus torazame,* whale shark *Rhincodon typus*, great white shark *Carcharodon carcharias*, zebra shark *Stegostoma tigrinum*, white-spotted bamboo shark *Chiloscyllium plagiosum,* brownbanded bamboo shark *Chiloscyllium punctatum,* grey bamboo shark *Chiloscyllium griseum,* epaulette shark *Hemiscyllium ocellatum,* shortfin mako shark *Isurus oxyrinchus,* spiny dogfish *Squalus acanthias*. Schematic phylogeny of the representatives of Chondrichthyes included in the analysis is presented at Fig. 1.

**Figure 2:**
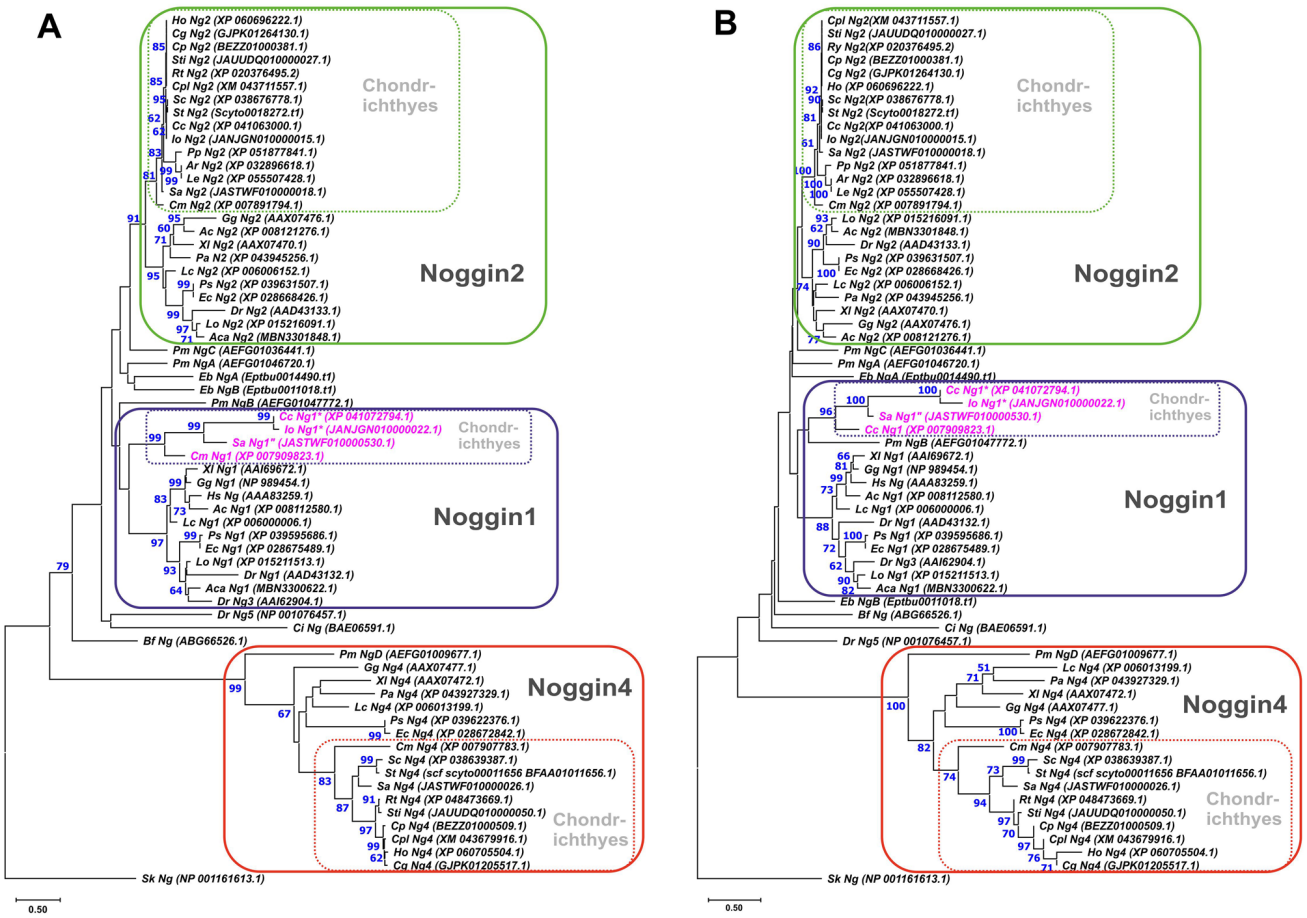
ML (**A**) and NJ (**B**) phylogenetic trees of vertebrate Noggin proteins. Bootstrap values > 50 are shown. Ac—*Anolis carolinensis,* Aca—*Amia calva*, Ar—*Amblyraja radiata*, Bf—*Branchiostoma floridae*, Cc—*Carcharodon carcharias*, Cg—*Chiloscyllium griseum*, Cp—*Chiloscyllium punctatum*, Cpl—*Chiloscyllium plagiosum*, Cm—*Callorhinchus milii*, Ci—*Ciona intestinalis*, Dr—*Danio rerio*, Eb—*Eptatretus burgeri*, Ec—*Erpetoichthys calabaricus*, Gg—*Gallus gallus*, Ho—*Hemiscyllium ocellatum*, Hs– *Homo sapiens*, Io—*Isurus oxyrinchus*, Lc—*Latimeria chalumnae*, Le—*Leucoraja erinacea,* Lo—*Lepisosteus oculatus*, Pa—*Protopterus annectens*, Pm—*Petromyzon marinus*, Pp—*Pristis pectinata*, Ps—*Polypterus senegalus*, Rt—*Rhincodon typus*, Sa—*Squalus acanthias,* Sc—*Scyliorhinus canicula*, Sk—*Saccoglossus kowalevskii,* St—*Scyliorhinus torazame*, Sti—*Stegostoma tigrinum*, Xl—*Xenopus laevis*.

Phylogenetic analysis shows that Noggin proteins of Chondrichthyes generally cluster with homologues of other gnathostomes, although in all cases, they tend to form subgroups on the branches of individual Noggin branches (Fig. 2). Multiple alignment of chondrichthyans’ and some other gnathostomes’ Noggins is shown at Supplementary Fig. 1S.

Among the analysed representatives of chondrichthyans, homologues of *noggin1* was found only in the genomes of the elephant shark (*C. milii*) and spiny dogfish *Squalus acanthias* (Supplementary File 1). *Squalus acanthias* Noggin1 contains a mismatch in conservative positions near the C-end (V instead I of all other Noggin1 from lancelet to human) (Supplementary Fig. 2S). Also we didn’t found the mRNA of this gene in the *S. acanthias* transcriptome sequences (https://www.ncbi.nlm.nih.gov/bioproject/PRJEB14721), where only *noggin2* and *noggin4* are presented. In a reduced form of a pseudogene were found in the shortfin mako shark *I. oxyrinchus* and the great white shark *C. carcharias* genomes. The *noggin1* pseudogene of *I. oxyrinchus* is located on Scaffold_22 and has stop codons in its open reading frame (ORF) (JANJGN010000022.1:4229860-4230732). The *noggin1* pseudogene of *C. carcharias* is located on chromosome 22 and also has stop codons in ORF (JAGDEE010000072.1:65581332-65581823). The same gene is present in the database with Gene ID: 121293691 as a two-exon version. This splicing variant theoretically makes it possible to obtain a shortened Noggin1 protein without stop codons; however, all genes of the Noggin family described to date in vertebrates are single-exon genes. The absence of functional Noggin1 in *C. carcharias* is indirectly confirmed by the fact that we were unable to detect *noggin1* cDNA fragments in the EST and transcriptome databases.

At the phylogenetic tree chondrichthyan Noggin1 proteins cluster closer to Noggin1 of gnathostomes but not very confidently (bootstran value is < 50) and form a subgroup within the Noggin1 clade. Noggin2 was found in all cartilaginous fish examined, and the Noggin2 cluster was quite confidently allocated to the Noggin2 branch of gnathostomes. Noggin4 was found in *C. milii*, *R. typus*, *S. canicula*, *S. torazame, C. plagiosum*, *C. punctatum, C. griseum*, *S. tigrinum, H. ocellatum* and *S. acanthias*. At the same time, Noggin4 was not found in the genomes of *C. carcharias*, *I. oxyrinchus*, *A. radiata*, *L. erinacea* and *P. pectinata*. The Noggin4 proteins of gnathostomes are very confidently clustered on a common branch, on which the chondrichthyan genes tend to move closer together.

In general, we can conclude from phylogenetic analysis that of the *noggin* genes described in gnathostomes, all three paralogues are present only in the most basally divergent branch—Holocephali. In the elasmobranchs examined, only *noggin2* is stably present. *Noggin4* has disappeared in some of the species examined, and surprisingly, *noggin1* is absent in most of elasmobranch genomes analysed (except for *S. acanthias* and pseudogenes preserved in *C. carcharias* and *I. oxyrinchus*).

To supplement the results of phylogenetic analysis and further test the disappearance of *noggin1* in elasmobranchs, an analysis of local genomic synteny of *noggins* in this clade and other gnathostomes was carried out. The main objectives of this analysis were to further test, using an independent criterion, the identified orthology of the *noggin* genes of chondrichthyans and other gnathostomes, as well as to confirm the absence of *noggin1* homologues in the vicinity of characteristic neighbouring genes.

**Figure 3:**
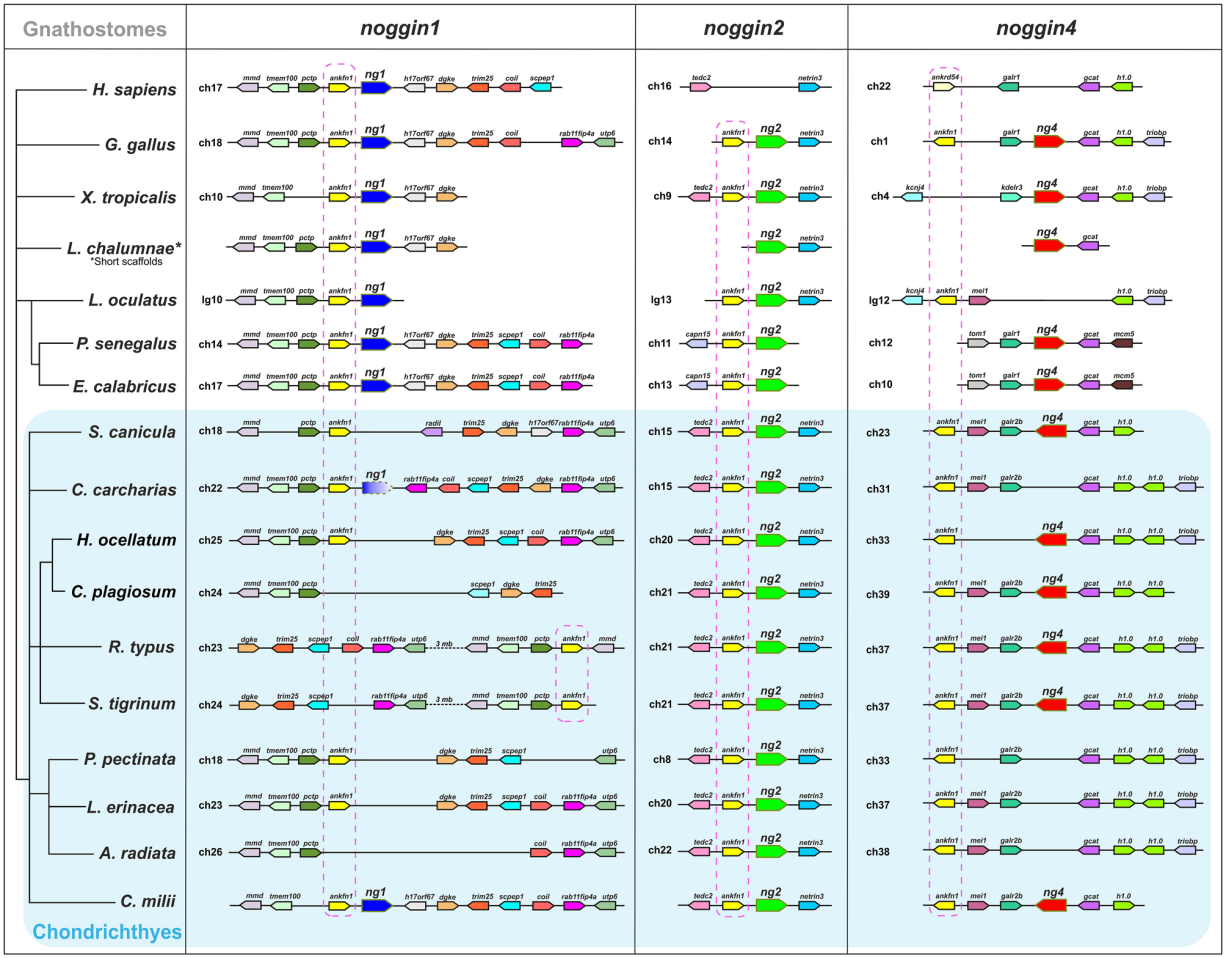
Local genomic synteny analysis of vertebrate *noggin* genes.

The analysis showed that one of the characteristic neighbouring genes for the *noggin* genes is the *ankfn1* gene (which encodes Ankyrin repeat and fibronectin type-III domain-containing protein 1), which was previously noted as a neighbour of the *noggin1/2* genes^31^. In this case, it is important that this gene was also found in the vicinity of the chondrichthyan *noggin4* gene (Fig. 3, dotted lines). This reflects the unity of origin of all three gnathostome *noggin*s. In addition to chondrichthyan *noggins*, the *ankfn1* gene is found in the vicinity of *noggin4* in birds (Fig. 3). We refer to all *ankfn1* paralogues here as "*ankfn1*" because they are registered as the same name in the genome of basally divergent Chondrichthyan representative *C. milii,* according to the gene IDs 103191444, 103178853, 103189246.

To establish the orthology of *noggins* in different groups of vertebrates, it is important to identify unique neighbouring genes for each of the *noggin* orthologues. The screening showed that such genes for *noggin2* are *netrin-3* and *tedc2* (which encode tubulin epsilon and delta complex protein 2) and for *noggin4* are *gcat* (glycine C-acetyltransferase), *galr2b* (galanin receptor 2b-like) and *mei1* (meiosis inhibitor protein 1). These neighbouring genes are found in the vicinity of the *Noggin* genes in all chondrichthyans examined and are also present in representatives of other groups of gnathostomes. Thus, based on a set of neighbouring genes, one can confidently identify the orthologous identity of the *noggin* gene in question. Additional confirmation of the disappearance of the *noggin4* genes in *C. carcharias*, *A. radiata* and *P. pectinata* is their absence in the syntenic region of the genome—between the *galr2b* and *gcat* genes, where it is located in all other representatives of chondrichthyans and other representatives of gnathostomes (Fig. 3).

The neighbourhood of *noggin1* is characterized by the presence of the following genes: in the 5' region, these genes are *tmem100* (transmembrane protein 100), *mmd* (monocyte to macrophage differentiation factor), and *pctp* (phosphatidylcholine transfer protein); in the 3' region, these genes are *h17orf67*, *dgke* (diacylglycerol kinase, epsilon), *trim25* (tripartite motif containing 25), *scpep1* (serine carboxypeptidase 1) and *coil* (coilin p80). The genome of any given animal does not necessarily contain the full set of these neighbours, but some of them are always present, and these genes (with the exception of *ankfn1*) are unique neighbours of *noggin1*. The disappearance of *noggin1* in chondrichthyans was confirmed by its absence between characteristic neighbouring genes in *A. radiata*, *P. pectinata*, *S. canicula* and *C. plagiosum*. *R. typus* and *S. fasciatum* show disruption of gene arrangement in the potential neighbourhood of *noggin1*. In the genome of *C. carcharias*, the *noggin1* pseudogene is located in the synteny region and has neighbouring genes characteristic of *noggin1*. In *C. milii*, all three *noggin* genes have gnathostome-specific neighbouring genes.

Thus, the results of the analysis of genomic synteny confirm the previous idea about the common origin of all three paralogues of the *noggin* genes of gnathostomes^31^. The phylogenetic analysis data were also confirmed, indicating the presence of *noggin2* orthologues in all chondrichthyans examined, the disappearance of *noggin4* in a number of representatives of the group, and the almost complete disappearance of *noggin1* in chondrichthyans (with the exception of the pseudogene preserved in *C. carcharias*). The fact that the disappearance of *noggin*s is observed only in representatives of elasmobranchs, while in the basally divergent group of chondrichthyan Holocephali, all three gnathostome *noggins* are present, indicates that the disappearance of *noggin1* in elasmobranchs has a secondary nature and may be the result of evolutionary specialization of representatives of this clade.

### Spatial expression of *noggin2* and *noggin4* in grey bamboo shark embryos

Since the absence of *noggin1* in elasmobranchs is unique for vertebrates, analysis of the expression pattern of the *noggin2* and *noggin4* present in this clade is of great interest. In particular, it would be interesting to evaluate whether *noggin2*, which, according to previous studies, is similar in properties to *noggin1* but differs significantly in expression pattern, can spatially compensate for the absence of *noggin1* in elasmobranchs.

Analysis of the expression pattern of *noggin2* and *noggin4* was carried out in embryos of the grey bamboo shark *C. griseum* by the whole mount in situ hybridization (ISH) method. To increase the specificity of the obtained signal we used stronger ISH conditions instead standard: temperature of probe hybridization was increased to 70 C, while the probe and AP antibodies concentrations were decreased twice against standard (see Material and Methods for details).

**Figure 4:**
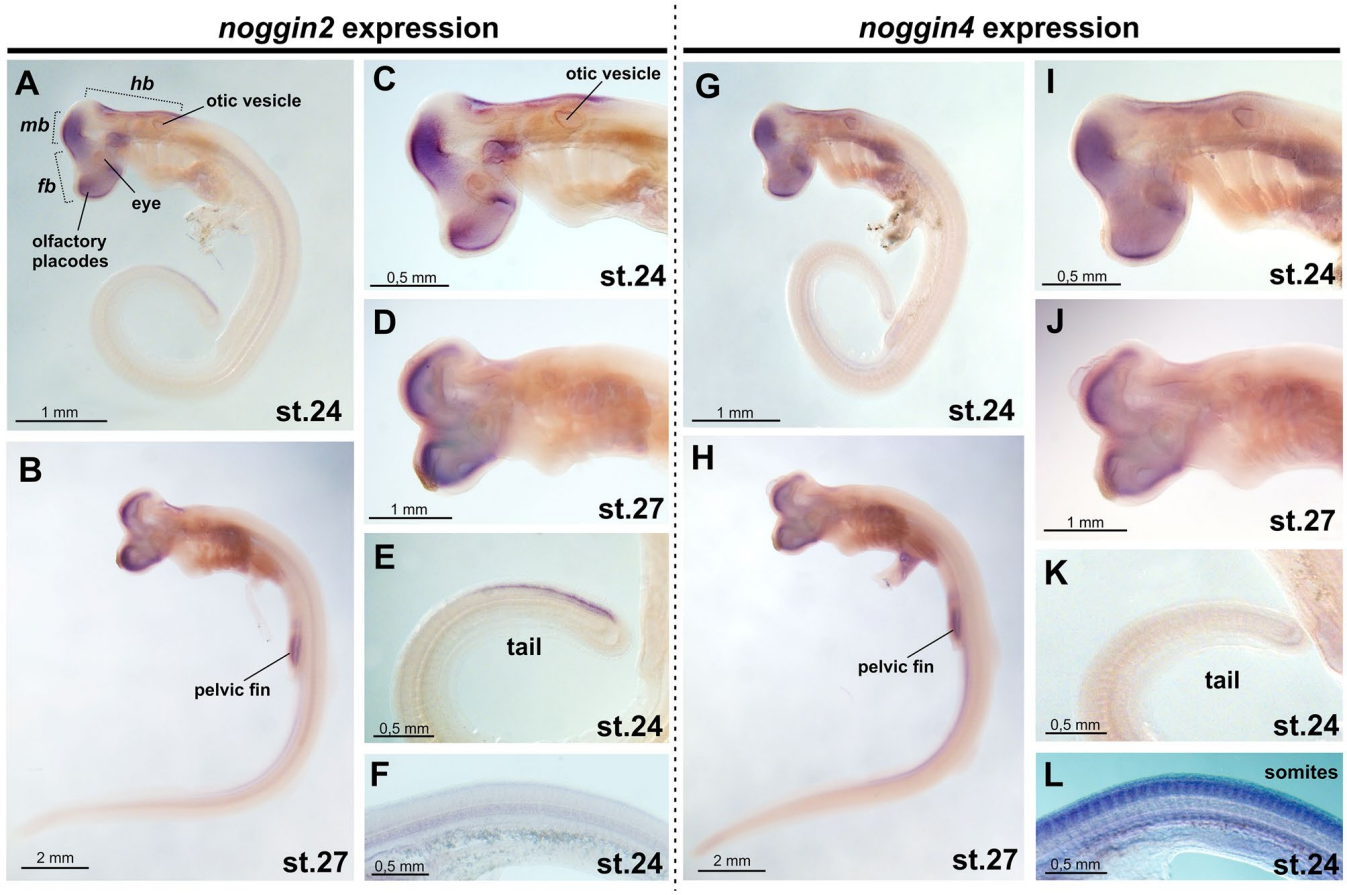
Spatial expression of *noggin2* (**A**–**F**) and *noggin4* (**G**–**L**) in the embryos of the grey bamboo shark *C. griseum*. *Fb* forebrain, *mb* midbrain, *hb* hindbrain.

**Figure 5:**
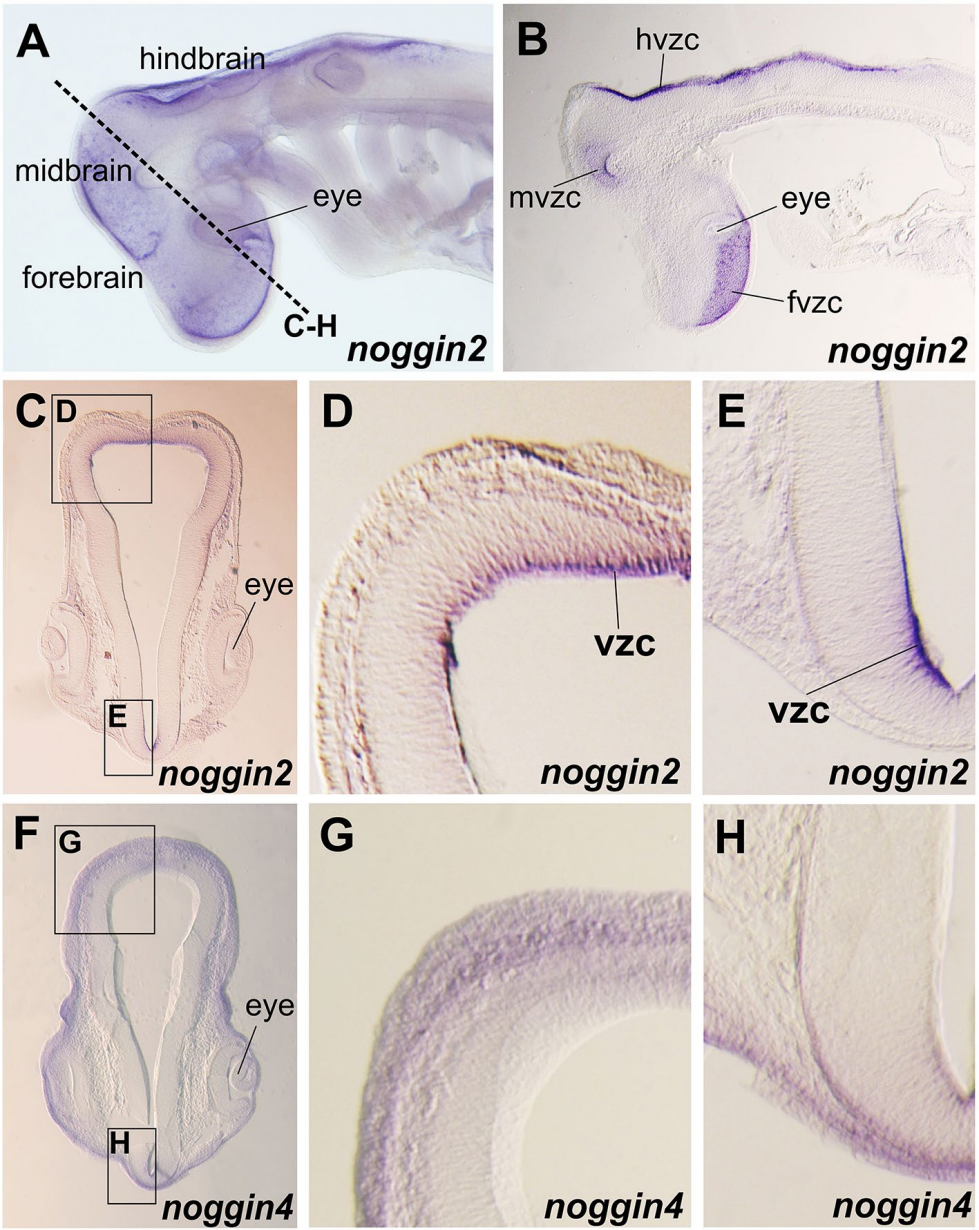
Sections of *C. griseum* embryos after *noggin2* (**A**–**E**) and *noggin4* (**F**–**H**) ISH. *fvzc* forebrain ventricular zone cells, *hvzc* hindbrain ventricular zone cells, *mvzc* midbrain ventricular zone cells. (**A**,**B**) stage 24, (**C**–**H**) stage 26.

In the brain region at stage 24 *noggin2* is diffusely expressed in telencephalon and mesencephalon, but is missing in the anterior part of diencephalon (Fig. 4A,C). As it is shown at sections (Fig. 5) *noggin2* is localized in an intense band of cells at the ventricular zone of forebrain, midbrain and hindbrain. Also *noggin2* expression is detected along the dorsal edge of the tail (Fig. 4E).

*Noggin4* at stage 24 is expressed diffusely in telencephalon, diencephalon and mesencephalon in head region (Figs. 4G,I; 5F–H). Unlike *noggin2*, *noggin4* is not detected along the dorsal edge of the tail (Fig. 4К). With longer incubation with AP substrate and under standard ISH conditions, *noggin4*, unlike *noggin2*, is detected in somites of the trunk and tail (compare Fig. 4L and F; Supplementary Fig. 3S).

At stage 27 *noggin2* and *noggin4* are expressed in the cells of ventricular zone of the telencephalon, diencephalon and mesencephalon (Fig. 4B,D,H,J). Also their expression is detected in pelvic fin buds.

As the observed diffuse patterns of *noggin2* and *noggin4* may raise questions regarding the specificity of the observed signals, we performed control ISH with *noggin2* and *noggin4* sense probes (Supplementary Fig. 4S). The almost complete absence of staining of these sense probes (except for some non-specific signal in the fourth ventricle) confirms the specificity of the observed expression patterns of *noggin2* and *noggin4* in *C. griseum* embryos.

Thus, *noggin2* and *noggin4* of *C. griseum* generally show an expression pattern similar to that of *noggin2* and *noggin4* orthologues in *D. rerio* and *Xenopus sp*^29,30,39^. The diffuse *noggin2* and *noggin4* expression was observed in head region of *X. laevis* (see Ref.^30^ and Fig. 3B,C,E,F).

### Shark *noggin2* induces complete secondary axes in *X. laevis*

**Figure 6:**
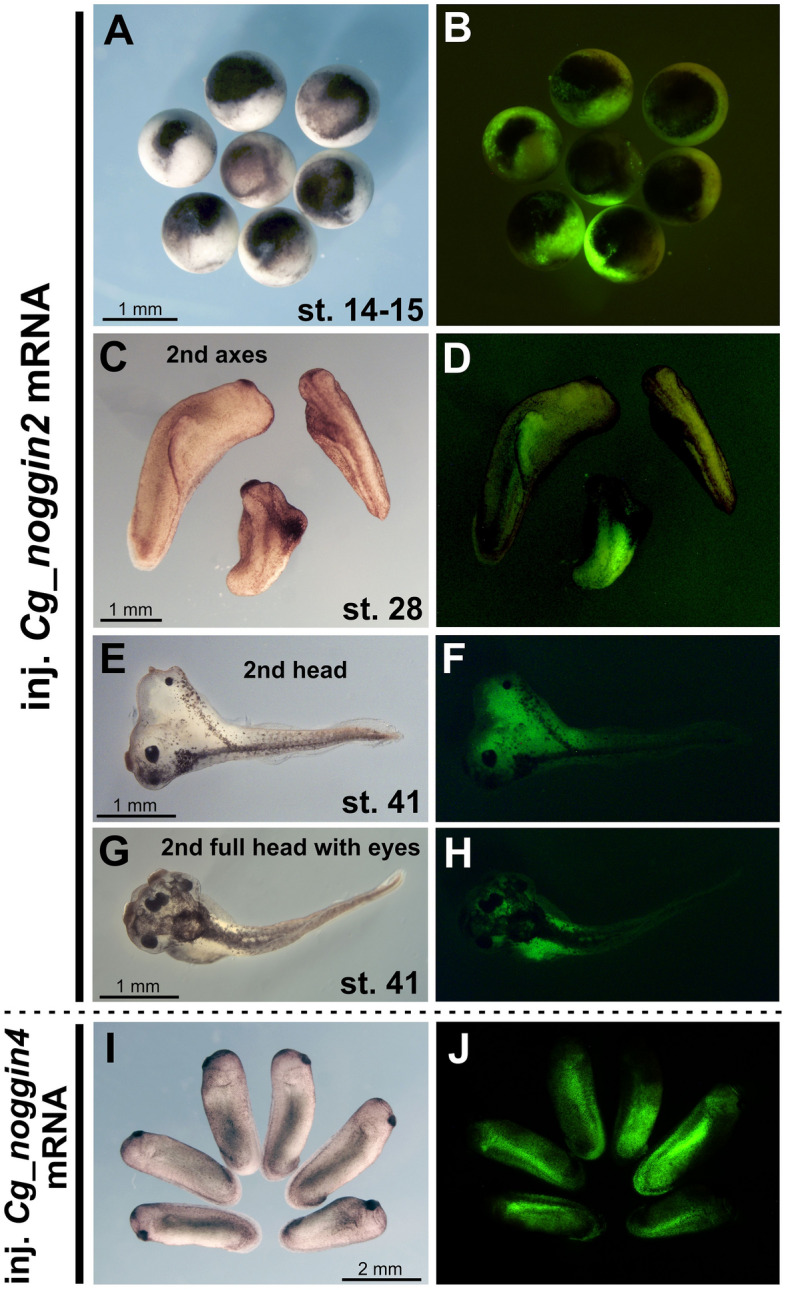
*C. griseum noggin2* mRNA induces the formation of additional body axes when injected into *X. laevis* embryos. (**A**,**B**) in high quantities (50 pg per embryo), *noggin2* mRNA of *C. griseum* causes disruption of normal development at the neurula stage and the formation of mushroom-shaped embryos. (**C**–**H**) injections of 5 pg per embryo of *C. griseum noggin2* mRNA lead to the induction of secondary body axes, including those containing full-fledged forehead structures and paired eyes. (**I**,**J**) *Noggin4* does not induce secondary axes in *X. laevis.*

Since functional experiments on shark embryos in vivo are difficult due to the structural features of their eggs and embryo development, testing of the functional activity of the *noggins* of chondrichthyan was carried out on amphibian (*X. laevis*) embryos. The ability of *noggin2* and *noggin4* of the grey bamboo shark *C. griseum* to induce secondary body axes in *X. laevis* embryos was assessed. To do this, synthetic grey bamboo shark *noggin2* and *noggin4* mRNAs were injected into the equatorial zone of the ventral region of *X. laevis* embryos at the 8-blastomere stage. As a result, it was found that 50 pg of shark *noggin2* mRNA injected into *X. laevis* embryos led to the disruption of normal development at the neurula stage and the formation of characteristic mushroom-shaped embryos (Fig. 6A,B). Similar phenotypic effects were observed when *X. laevis noggin2* mRNA was injected^18^. Injections of smaller amounts of *noggin2* mRNA (5 pg per embryo) resulted in the induction of additional body axes in 53% of cases (n = 250), including complete ones containing forehead structures and eyes in 8% of cases (Fig. 6C–F, Supplementary Fig. 5S). In some cases, the formation of full-fledged second heads with absolutely complete anterior cephalic regions and paired eyes was observed (Fig. 6G,H). Similar inductive activity has also been described for *X. laevis noggin2*.

Shark *noggin4* mRNA, similar to *X. laevis noggin4* mRNA, did not show the ability to induce the formation of secondary axes in *X. laevis* (Fig. 6I,J; Ref.^34^).

The results obtained demonstrate the conserved properties of the shark *noggin2* and *noggin4* genes.

## Discussion

### The absence of *noggin1* in elasmobranchs is a unique case for vertebrates

In the classical model of neural induction, Noggin1 is traditionally considered one of the main actors involved in early embryonic differentiation by inhibiting the activity of the BMP signalling pathway. In addition to its early inductive function, Noggin1 has been described to participate in a wide range of developmental processes^17^. The absence of *noggin1* in elasmobranchs demonstrated in the present work is unique for vertebrates and, apparently, reflects the features of early induction processes in the embryos of this evolutionary branch.

The absence of *noggin1* in elasmobranchs is obviously secondary, since the basally divergent group of chondrichthyan—Holocephalii—has all three *noggin* genes, found and described in other gnathostomes—*noggin1*, *noggin2* and *noggin4*. In addition, noggin1 DNA was found in genome of spiny dogfish *S. acanthias*. However, the expression of *S. acanthias noggin1* and its functional viability raise questions. We didn't find the mRNA of this gene in the transcriptome databases (transcriptome shotgun assemblies), where only *S. acanthias noggin2* and *noggin4* are represented. Also, aminoacid sequence of *S. acanthias* Noggin1 contains a mismatch in conservative positions near the C-end (V instead I) distinguishing it from all other Noggin1 from lancelet to human (Supplementary Fig. 2S). Just upstream of this I to V mismatch, *S. acanthias* Noggin1 contains the inserted sequence PHRDREPHRD, which is unique to this protein. It was shown that C’-region is important for functional activity of Noggin proteins and Noggin4 contain R instead I in this position, that correlates with their inability to bind and inhibit BMP signal (Supplementary Fig. 1; Ref.^34^). Such V > I mismatch and insertion upstream it are unique for *S. acanthias* Noggin1 and are not observed in other noggins from lancelet to humans (Supplementary Fig. 2). The *noggin1* pseudogene with stop codons inside the ORF were found in genomes of shortfin mako shark *I. oxyrinchus* and the great white shark *C. carcharias*.

As shown by the analysis of local genomic synteny, all three gnathostome *noggin* paralogues have a common origin. Moreover, each of the gnathostome *noggins* has a number of unique nearby genes that are conserved in different groups of vertebrates. These findings allow one to confidently identify (and distinguish) *noggin* genes by analyzing their local genomic synteny. The absence of *noggin1* in elasmobranchs is confirmed by such synteny analysis, according to the results of which *noggin1* is absent in the genome regions, localized and determined by the presence of specific nearby genes.

In our opinion, the discovery of *noggin1* sequences (including partial sequences) in some representatives of Chondrichthyes presents an interesting picture when compared with the phylogenetic tree of the clade. *Noggin1* is found in genome of *C. milii*, a member of the basally divergent group of chondrichthyans, sister to the elasmobranchs. In addition, complete *noggin1* DNA (with some with unique features discussed above) was found in the genome of the spiny dogfish *S. acanthias*, a member of the order Squaliformes, one of the basally divergent lineages of sharks^40^. This suggests that the loss of *noggin1* in most of the elasmobranchs studied was secondary. *Noggin1* pseudogenes were found in genomes of *I. oxyrinchus* and *C. carcharias*, which belong to the order Lamniformes and are phylogenetically close to each other^40^. Moreover, the discovery of partial *noggin1* sequences in the genomes of some sharks in itself indicates the sensitivity of the used search algorithms and increases the reliability of "negative data" about the absence of *noggin1* in the majority of species analyzed.

### Genes* noggin2* and *noggin4* are conserved in vertebrates both in their expression patterns and functional properties

The expression patterns of *noggin2* and *noggin4* observed in *C. griseum* show similarities with previously described patterns of their orthologues in fish and amphibians. In *D. rerio*, *noggin2* expression has been described in the forebrain and tail bud (with greater intensity in its ventral region) during different growth stages^39^. In *X. tropicalis* and *X. laevis noggin2* is expressed in both the anterior and posterior regions of the brain, as well as in the heart region^29,30^. Relatively weak expression in somites was also noted. At the same time, a characteristic feature of the *noggin1* pattern in *Xenopus* and *Danio* is expression in axial structures and mesodermal derivatives, starting from their formation at the gastrula stage. At pharyngula stages, expression was observed in the notochord, neural tube, paraxial mesoderm, axial mesendoderm and pharyngeal arches^29,39^.

In *C. griseum*, we observed *noggin2* expression in the brain, growing tail bud, and heart region. However, we did not detect *C. griseum noggin2* in the notochord and axial mesoderm. This allows us to conclude that the expression pattern of *noggin2* in *C. griseum* is consistent with the expression of its orthologues in other vertebrates. *Noggin4* in *C. griseum* shows diffuse expression in the head structures, pharyngeal arches, and trunk and tail somites. Such a pattern also corresponds to the previously described *noggin4* expression in *X. laevis*^30^.

The functional properties of the *noggin* genes of *C. griseum* correspond to the previously described properties of their homologues in other gnathostomes. *Noggin2* of *C. griseum* showed the ability to induce secondary body axes in *X. laevis* embryos, while *noggin4* showed no such inductive ability. The observed ability of shark *noggin2* to induce complete secondary axes containing forehead structures and paired eyes suggests its ability to suppress the activity of at least the BMP and Wnt signalling pathways^37,38^. The inability of *noggin4* to induce the axis may be explained by the features of the primary amino acid sequence of its protein, which contains a number of substitutions conserved for Noggin4, disrupting the ability of these proteins to bind BMP molecules and modulate the activity of the BMP signalling pathway (Fig. S1 in Ref.^34^^,^^41^).

Thus, in the absence of the *noggin1* gene, the *noggin2* and *noggin4* genes of *C. griseum* exhibit properties conserved among their orthologues. At the same time, similarities in the patterns and functional properties of shark *noggin2* and *noggin4* are observed not only with the orthologues of gnathostomes but also with the *noggins* of lampreys^31^, which reflects the conservatism of the *Noggin* family in vertebrates in general.

### The loss of *noggin1* may be associated with structural features of the cartilaginous skeleton of elasmobranchs

The disappearance of the *noggin1* gene in elasmobranchs may be associated with the structural features of both the embryo and adult animals. The role of Noggin1 as one of the key early neural inducers has been demonstrated in amphibians. However, it has not been clearly identified in other vertebrates. In *D. rerio* embryos, unlike in *Xenopus*, *noggin1* at the gastrula stage is not expressed in the primary organizer region and therefore is not involved in the early axial differentiation of the embryo^42^. In chick embryos, which are similar to shark embryos in the ratio of yolk volume to embryo size, *noggin1* is expressed in the area of Hensen’s node (organizer), but no evidence of its functional role in early differentiation of the embryo has been obtained^43^. These data indicate that the roles of Noggin1 in neural induction may differ among different classes of vertebrates. It is possible that the early function of noggin1 could be replaced by other inducers. It has recently been discovered that, at least in *X. laevis*, weak expression of *noggin1* and *noggin2* occurs in cells of the early Spemann organiser^44^. The authors also showed that Noggin2 plays an important role in determining the size of the notochord primordium. This suggests that elasmobranchs may be able to replace the function of the lost *noggin1* in the organizer cells. To test this hypothesis, it would be necessary to analyze the expression of *noggin2* in elasmobranchs at appropriate early stages of embryogenesis in the future. However, carrying out such studies, as well as direct functional experiments on early elasmobranch embryos, remains technically difficult because the representatives of this group available to us undergo internal fertilization, after which the embryos develop in eggs with a dense opaque shell in seawater. We can therefore only draw analogies with data from other vertebrate groups when studying the properties and role of *noggin* genes in ontogeny.

The main function of Noggin is traditionally considered to be inhibition of the BMP signalling pathway involved in the formation of connective tissues and the internal skeleton of vertebrates. The skeleton of elasmobranchs differs from the skeletons of other classes of vertebrates in that it consists almost entirely of cartilaginous tissue, hyaline-like cartilage, throughout the life of the adult animal^45^. Moreover, this skeletal condition is considered secondary, possibly resulting from a premature stop in the formation of endochondral ossifications characteristic of vertebrates^46^. In this case, the cartilaginous skeleton can be strengthened due to surface mineralization through the formation of “tesserae”—hexagonal plates that form the calcified shell of the skeletal elements. The absence of the stage of chondrocyte hypertrophy during the formation of cartilage in elasmobranchs and the structural features of the tesserae indicate that calcification of the cartilaginous skeleton is an evolutionarily independent direction in the development of biomineral-based skeletal reinforcement^46–48^. It can be noted that this unique method of formation of the internal cartilaginous skeleton in elasmobranchs correlates with the absence of *noggin1* as one of the factors involved in the regulation of the development of cartilage and bone tissue.

The link between Noggin1 activity and chondrogenesis in vertebrates has been established previously. In *D. rerio noggin1* was shown to be expressed at later stages in presumptive cartilage cells in the branchial arches, head skeleton and pectoral fin buds^42^. The authors suggested that expression in developing cartilage may reflect the ancestral state/function of *noggin1*, which is later co-opted by early organizer and axial mesoderm functions^42^. Expression of the zebrafish *noggin3* paralogue (closest to *noggin1*) in the formation of neurocranium and pectoral cartilage was also observed^39^. The expression of *noggin* in chondrocytes and its involvement in chondrogenesis have been demonstrated in mice^23^. The onset of *noggin1* expression in mice has been noted since the beginning of skeletal development in the core regions of cell condensation in the limb and trunk, suggesting its important role in early skeletal development^49^. Noggin-null mutant mice show severe cartilage dysplasia and joint defects^23^. Overexpression of *noggin1* under the control of the collagen promoter, causing suppression of the BMP signal, led to a significant reduction in cartilage tissues and structures. This indicates the importance of the BMP signal for the development of cartilage tissue^28^. Similar results were obtained with retroviral expression of *noggin1* in the developing avian limb, where BMP inhibition also resulted in impaired chondrocyte differentiation and chondrogenesis^50^. In *Xenopus* tadpoles the overexpression of *noggin1* at stages 50–51 results in a reduction in the number of digit cartilage condensations^51^. On the other hand, it has been shown that in *noggin* null mice a delay or suppression of ossification of some bones is observed, indicating that increasing the level of BMP signalling can not only increase but also reduce the level of ossification depending on the location and embryonic origin of the bones^52^.

Taken together, these data indicate the involvement of the BMP/Noggin regulatory loop in the formation of the osteochondral skeleton in vertebrates. It can be assumed that the emergence and consolidation in elasmobranchs evolution of their unique histological characteristic skeleton could be associated with a shift in the BMP/Noggin regulatory balance in this group due to the disappearance of Noggin1.

Thus, it is tempting to speculate that the emergence and consolidation of their unique histologically distinctive skeleton in elasmobranch evolution may be associated with a shift in the BMP/Noggin regulatory balance in this group due to the disappearance of *noggin1*. However, more research is needed to determine whether there is indeed a mechanistic link between the absence of *noggin1* in elasmobranchs and their lack of internal skeletal ossification. At the very least, if we accept the assumption that such a link does exist, it is necessary to explain why chimeras (basal group of chondrichthyans, sister to elasmobranchs), despite the presence of *noggin1* in their genome, also lack internal ossification of the cartilaginous skeleton. It can be speculated that, like elasmobranchs, chimeras lack *noggin1* expression in connective tissues, but for a different reason: the absence of specific enhancer elements that direct *noggin* expression to these tissues. To test this hypothesis, the expression pattern of *noggin1* in chimeras needs to be studied in more detail. Perhaps in sharks, using *noggin1* as an example, we can observe successive stages of the disappearance of an initially functional gene: from the weakening of gene expression/function (*C. milii* and *C. acanthias*), through its pseudogenisation (*C. carcharias* and *I. oxyrinchus*), to its complete disappearance from the genome (most of the elasmobranch species analysed).

## Materials and methods

### Animals and samples preparation

All animal experiments were approved by the Shemyakin-Ovchinnikov Institute of Bioorganic Chemistry (Moscow, Russia) Animal Committee. Experimental protocols of ISH and in vivo injections were carried out in accordance with Shemyakin-Ovchinnikov Institute of Bioorganic Chemistry (Moscow, Russia) Animal Committee Protocols. The study was carried out in accordance with the ARRIVE guidelines.

*C. griseum* eggs and embryos were collected in collaboration with the scientific department of the Moskvarium Center for Oceanography and Marine Biology (Moscow, Russia).

The embryos of *C. griseum* were staged in accordance with Ballard et al. 1993^53^, *X. laevis* were staged after Nieuwkoop and Faber 1994^54^.

For ISH, embryos were fixed in MEMFA solution (3.7% formaldehyde, 100 mM MOPS, 2 mM EGTA, 1 mM MgSO4), dehydrated in methanol and kept at − 20C.

*C. griseum* total RNA samples were obtained from stage 26 lysed embryos by purification with the Analytic Jena innuPREP RNA Mini Kit 2.0 (REFINE KIT).

### Phylogeny and synteny analyses

The search for homologs was carried out in Blastn (https://blast.ncbi.nlm.nih.gov/Blast.cgi?PROGRAM=blastn&PAGE_TYPE=BlastSearch&BLAST_SPEC=&LINK_LOC=blasttab&LAST_PAGE=blastn) and tBlastn (https://blast.ncbi.nlm.nih.gov/Blast.cgi?PROGRAM=tblastn&PAGE_TYPE=BlastSearch&BLAST_SPEC=&LINK_LOC=blasttab&LAST_PAGE=blastn) sections. We checked available Nucleotide collections (nr/nt) and whole-genome shotgun contigs (wgs).

Multiple alignment was performed by ClustalW algorhythm in the MEGA11 program.

Phylogenetic analyses of Noggin protein sequences were performed via the Maximum Likehood (ML) and Neighbor-Joining (NJ) methods using the MEGA11 program^55^.

The choosing of optimal model was made in MEGA11. The results are present in Supplementary Table 1.

In ML method JTT matrix-based model^56^ with frequencies and Gamma distribution was used. The percentage of trees in which the associated taxa clustered together in the bootstrap test (500 replicates) is shown next to the branches^57^. The tree is drawn to scale, with branch lengths measured in the number of substitutions per site. This analysis involved 66 amino acid sequences. There were a total of 413 positions in the final dataset.

In NJ method analysis^58^ the optimal tree is shown. The percentage of replicate trees in which the associated taxa clustered together in the bootstrap test (500 replicates) are shown next to the branches^57^. This analysis involved 66 amino acid sequences. All ambiguous positions were removed for each sequence pair (pairwise deletion option). There were a total of 413 positions in the final dataset.

Synteny analysis and search for neighboring genes were also carried out on the NCBI website (https://www.ncbi.nlm.nih.gov/).

Noggin amino acid multiple Alignment shown at Figs. S1 and S2 were performed by Clustal Omega.

### *C. griseum Noggin2* and *Noggin4* cDNAs, ISH, functional tests

Full-length *C. griseum Noggin2* and *Noggin4* cDNAs for ISH and functional experiments were generated using nested PCR with the following primer pairs:

Cg_Ng2_full_Frw1: CCGAACTGGCCCGTTTAAAA.

Cg_Ng2_full_Rev1: CTGCATGAGAACATTTCTCC.

Cg_Ng2_full_Frw2: AATGAATTCGCCACCATGGAGCTGCCACAGTATAT.

Cg_Ng2_full_Rev2: AATCTCGAGTTAACAGGAACACTTGCACT.

Cg_Ng4_full_Frw1: AGGTGACGGACAACGGCGCA.

Cg_Ng4_full_Rev1: TGAACAGCCAGCAGGATGGC.

Cg_Ng4_full_Frw2: AATGAATTCGCCACCATGCCTCGGGAGCTCCCCC.

Cg_Ng4_full_Rev2: AATCTCGAGTCACCGACAGGAGCACTTGC.

In the first round of PCR (30 cycles), primers Frw1 and Rev1 were used. The resulting PCR product was purified and used as a template in the next round of PCR (20 cycles) with primers Frw2 (which contains Kozak sequence and start ATG) and Rev2. PCR was performed with Encyclo polymerase Evrogen kit (www.evrogen.ru).

The resulting cDNA fragments were cloned into the pAL2-T vector (Evrogen) and cDNA inserts of 3 clones of each *Noggin* were sequenced. To obtain mRNA for injection, *Noggin2* and *Noggin4* cDNAs were recloned into the pCS2 vector. mRNA synthesis was carried out by SP6 mMessage mMachine kit (Thermofisher).

ISH was carried out according to the protocol according to^18,31,59,60^ with minor changes in order to increase the signal specificity. The probe concentrations were 500 ng/ml of PH buffer (vs. standard 1000 ng/ml), the temperature of hybridization was 70 C (vs. standard 65 C) and the concentration of AP antibodies was 1: 4000 (vs. standard 1:2000).

Injections of synthetic *Noggin2* and *Noggin4* mRNAs into *X. laevis* embryos were carried out at the 8-blastomere stage in the ventral equatorial region. To visualize the distribution of the injected material in the embryo, the fluorescent dye fluorescein lysin dextran was added to the mixture.

Agarose sections 30–40 μm were performed as^61^.

Photography was carried out using a Leica M205 stereo microscope.

### Supplementary Information

Supplementary Figures.

## Data Availability

For genome synteny analysis we compared the Noggins’ nearby genes in the available genomic sequences of the following representatives of Elasmobranchs: Holocephali, sister group to Elasmobranches: *Callorhinchus milii* (elephant shark)—https://www.ncbi.nlm.nih.gov/datasets/genome/GCF_018977255.1/. Elasmobranches: Ray: *Pristis pectinata* (smalltooth sawfish)—https://www.ncbi.nlm.nih.gov/datasets/genome/GCF_009764475.1/. Skates: *Amblyraja radiata* (thorny skate, skate)—https://www.ncbi.nlm.nih.gov/datasets/genome/GCF_010909765.2/. *Leucoraja erinacea* (little skate)—https://www.ncbi.nlm.nih.gov/datasets/genome/GCF_028641065.1/. Sharks: *Squalus acanthias* (spiny dogfish)—https://www.ncbi.nlm.nih.gov/datasets/genome/GCA_030390025.1/. *Scyliorhinus torazame* (cloudy shark)—https://www.ncbi.nlm.nih.gov/datasets/genome/GCA_003427355.1/. *Scyliorhinus canicula* (smaller spotted catshark)—https://www.ncbi.nlm.nih.gov/datasets/genome/GCF_902713615.1/. *Rhincodon typus* (whale shark)—https://www.ncbi.nlm.nih.gov/datasets/genome/GCF_021869965.1/. *Carcharodon carcharias* (great white shark)—https://www.ncbi.nlm.nih.gov/datasets/genome/GCF_017639515.1/. *Stegostoma tigrinum* (zebra shark)—https://www.ncbi.nlm.nih.gov/datasets/genome/GCF_022316705.1/. *Chiloscyllium plagiosum* (whitespotted bambooshark)—https://www.ncbi.nlm.nih.gov/datasets/genome/GCF_004010195.1/. *Chiloscyllium punctatum* (brownbanded bamboo shark) https://www.ncbi.nlm.nih.gov/datasets/genome/GCA_003427335.1/. *Isurus oxyrinchus* (shortfin mako shark) https://www.ncbi.nlm.nih.gov/datasets/genome/GCA_026770705.1/. *Hemiscyllium ocellatum* (epaulette shark) https://www.ncbi.nlm.nih.gov/datasets/genome/GCF_020745735.1/. Representatives of bony fishes (*Lepisosteus oculatus* and *Latimeria chalumnae*), as well as tetrapods (*Xenopus tropicalis*, *Gallus gallus*, *Homo sapiens*) were also included in the analysis.
