# Supplementary material for: Loss of noggin1, a classic embryonic inducer gene, in elasmobranchs
Source: Sci Rep. 2024 Feb 15;14:3805. doi: 10.1038/s41598-024-54435-9 (PMC10869764; doi:10.1038/s41598-024-54435-9)
Supplement: Supplementary file 1 — Supplementary Figures. [file 41598_2024_54435_MOESM1_ESM.docx]

**Loss of *noggin1*, a Classic Embryonic Inducer Gene, in Elasmobranchs**

Galina V. Ermakova^1^, Irina V. Meyntser^2^, Andrey G. Zaraisky^1,3*^, Andrey V. Bayramov^1*^

**SUPPLEMENTARY MATERIAL**

**........10........20........30........40........50........60........70........80........90..
Hs_Ng(AAA83259.1) --------------------------------MERCPSLGVTLYALVVVLGLRATPAGGQHYLHIRPAPSDNLPLVDLIEHPDPIFDPKEKD
Gg_Ng1(NP_989454.1) --------------------------------MDHSQCL-VTIYAAAVLLGLRLQQGSCQHYLHIRPAPSDNLPLVDLIEHPDPIFDPKEKD
Xl_Ng1(AAI69672.1) --------------------------------MDHSQCL-VTIYALMVFLGLRIDQGGCQHYLHIRPAPSENLPLVDLIEHPDPIYDPKEKD
Cc_Ng1*(XP_041072794.1) ---------------------------------------MLTLYLG-----LWSRSVSGQHYLQLRPAATQHPPLLELWERPDPELDPKDSD
Cm_Ng1(XP_007909823.1) ---------------------------------MDLSRRRLTVYLATLLLCVWVRLGAGQHYLHLRPSPSDHLPLLELLEPPDPDLDPKDKD
Gg_Ng2(AAX07476.1) --------------------------------MTAIG---ALLLCSCLGLLR-P--GAGQPFLRLRPSPSDNLPVKDIVEHPDPEYDPKEQD
Xl_Ng2(AAX07470.1) --------------------------------MKRINLPEAFLLCLWLFLVQHQ--GCCQPYLRLRPSPSENLPVKDIIEHPDPEQDPKEQD
Sc_Ng2(XP_038676778.1) -----------------------------------MELPQYMLSCFCMLLLQH---GLCQPYLHLRPVPSDSLPVVDIIEHPDPDNDPKDND
Ry_Ng2(XP_020376495.2) -----------------------------------MELPQYMLSCFCMLLLQH---GLCQPYLHLRPVPSDSLPVVDIIEHPDPDNDPKDND
Cp_Ng2(XM_043711557.1) -----------------------------------MELPQYMLSCFCMLLLQH---GLCQPYLHLRPVPSDSLPVVDIIEHPDPDNDPKDND
Ar_Ng2(XP_032896618.1) -----------------------------------MEPPHCLLSCLCVLLLRH---GLCQPYLHLRPVPSDSLPVLDIIEQPDPDSDPKDSD
Cm_Ng2(XP_007891794.1) -----------------------------------MEMPHYFLTGFCMLLLHQT--GLCQPYLRLRPAPSDSLPVVDIIEHPDPEYDPKEND
Cc_Ng2(XP_041063000.1) -----------------------------------MELPQYMLSCFCMLLLQH---GLCQPYLHLRPVPSDSLPVVDIIEHPDPDNDPKDND
Gg_Ng4(AAX07477.1) ----MQDPCFYLLLILCLLPL------------PLGATGLEDPRDP-LPPP-LSTSDPTAHLLRGRPSAPVRPYSLSLSPE-DYRYAPKPRH
Xl_Ng4(AAX07472.1) --------MAHILFLSWLVTLG-----------TWGLTAICGQTND-LQHYNQTQKDMDIGSLRRRLSSGTRPYSLSLSPQ-DYHYSPKPKH
Sc_Ng4(XP_038639387.1) MAREFSYSGLALVSCYIFILHHCVKSSSPARGQSHSSDVLTGKAAIKLHPAPRPDSQAESAWVRMKASNTVRPYSLALTKD-HYHHSPRLKH
Rt_Ng4(XP_048473669.1) MPREVSHTVATLFPCQAIIVLLLVQSGLLAQPRSHSPDAQAGRPGIRLRPTPRPGSEAESAWSRMKASNSVRPYSLTLSDD-HYRYSPRPKH
Cp_Ng4(XM_043679916.1) MPRELPRTAATPFPCQAIIVFLLLHSGSSAQPRSRSADAQAGRPGTRLRPTPRPGSEAETAWGRMKVSNSVRPYSLSLSDH-HYRYSPRPKQ
Cm_Ng4(XP_007907783.1) ------MSWPAVLCCLSLLAWY---------PPARSLRIRPGRKGS---ATPKPVAEVESAWIRMRASSAVRPYSLSLNKD-HYHYFPGAQH**

**.....100.......110.......120.......130.......140.......150.......160.......170.......180....
Hs_Ng(AAA83259.1) LNETLLRSLLGGHYDPGFMATSPPEDRPGGGGGAAGGAEDLAELDQLLRQRPSGAMPSEIKGLEFSEG-LAQGKKQRLSKKLRRKLQMWLWS
Gg_Ng1(NP_989454.1) LNETLLRSLMGGHFDPNFMAMSLPEDRLG--------VDDLAELDLLLRQRPSGAMPGEIKGLEFYDG-LQPGKKHRLSKKLRRKLQMWLWS
Xl_Ng1(AAI69672.1) LNETLLRTLMVGHFDPNFMATILPEERLG--------VEDLGELDLLLRQKPSGAMPAEIKGLEFYEG-LQS-KKHRLSKKLRRKLQMWLWS
Cc_Ng1*(XP_041072794.1) LDEVTLRLKLARDLDPDFMSPRFPG------------------PEP---------VP-----------------------------------
Cm_Ng1(XP_007909823.1) MDETTLRKKLAANFDPNFMALRLPEGW----------EPEAGAPEPGLRHRPGGFMPNHIRRVDFVV---AAGRKQRLSKKLRRRLQLWLWS
Gg_Ng2(AAX07476.1) LDERTLRKKLGSHFDPGFMAVAVPG------------PANASAGAEAAAGRAR---AAELRWLER------GGPRLRVGKKARRKVLQWLWA
Xl_Ng2(AAX07470.1) LDERTLRKKLGSNFDPNFMSVVLPN------------SVNTSTQDSLTKMKTLGSIPLELKKLDLSE--TPYGDRIRMGKKARRKFLQWLWA
Sc_Ng2(XP_038676778.1) LDERTLRKKLGSHFDPNFMSITLPQ------------PEDSQSRDPNSRFKPLGAMPNDIKRLDLSE--TPYGRKMKLGKKARRKFQQWLWS
Rt_Ng2(XP_020376495.2) LDERALRKKLGSHFDPNFMSITLPQ------------LDDSPSRDPNSRFKPLGAMPNDIKRLDLSE--TPYGRKMKLGKKARRKFQQWLWS
Cp_Ng2(XM_043711557.1) LDERALRKKLGSHFDPNFMSITLPQ------------LDDSPSRDPNSRFKPLGAMPNDIKRLDLSE--TPYGRKMKLGKKARRKFQQWLWS
Ar_Ng2(XP_032896618.1) LDERTLRKRLGSHFDPGFMSITLPD------------ADPSAPRDPGGRLKPLGALPNDIKRLDVSE--TPYGRRMKLGKKARRRLQQWLWS
Cm_Ng2(XP_007891794.1) LDERTLRKKLGSHFDPSFMSVTLPE------------GEAFTPRDPNSRFKPLGAMPNDIKRLNLSE--TPYGRKMKLGKKARRKFQQWLWS
Cc_Ng2(XP_041063000.1) LDERTLRKKLGSHFDPNFMSITLPQ------------LEDSPSRDPNSRFKPLGAMPNDIKRLDLSE--TPYGRKMKLGKKARRKFQQWLWS
Gg_Ng4(AAX07477.1) LRPGRLRRLLGPAFDPFWMSSEE---PLGRNLSAKEDLETMSRELADSSGRYRRKLWREVEG-----MELPPELPAD----MARLLRRWLVE
Xl_Ng4(AAX07472.1) LRVSRLLRLLGSSFDPFWMSVEQ---PADN---GTSLLSTLSQDIYDGASRYRKKLSQEAQALDFDSLQLPTELSANSSQHIQNEIRQWLVQ
Sc_Ng4(XP_038639387.1) LNARKLMKLLGSSFDPFWMSVK----SRRGNKSHEG-LTLLNRDLAAGAVRSRRKLWQEAQRLDVG-ISPAGRAEGNVTEAGRAQWKQWLVE
Rt_Ng4(XP_048473669.1) LDSKRLRKLLGPAFDPFWMSVT----GRHRNESERDDLLQLSSELAAAALRLRGKLWQEARRLEP---------AGMASEAAKAQWRRWMVR
Cp_Ng4(XM_043679916.1) LDPKRLRKLLGPSFDPFWMSVT----GRHGNESERGHLLQLSGELAAAALRLRGKLWHEAQRLEP---------AAVASEADKAQWRRWLVR
Cm_Ng4(XP_007907783.1) LNPRRLQKLLGSNFDPFWMAVDPPDGAGRGNESHAD-LVSRSPRLTEPANRYQKKLRHEAERLGL----------GAGGDGGKEALARWLVK**

**...190.......200.......210.......220.......230.......240.......250.......260.......270...
Hs_Ng(AAA83259.1) QTFCPVLYAWNDLGSRFWPRYVKVGSCF-SKRSCSVPEGMVCKPSKSVHLTVLRWRCQRRGG-----QR-CGWIPIQYPIISECKCSC-
Gg_Ng1(NP_989454.1) QTFCPVLYTWNDLGSRFWPRYVKVGSCY-SKRSCSVPEGMVCKPAKSVHLTILRWRCQRRGG-----QR-CTWIPIQYPIIAECKCSC-
Xl_Ng1(AAI69672.1) QTFCPVLYTWNDLGTRFWPRYVKVGSCY-SKRSCSVPEGMVCKAAKSMHLTILRWRCQRRVQ-----QK-CAWITIQYPVISECKCSC-
Cc_Ng1*(XP_041072794.1) -----------------------------------------------------RWD---RER-----H--CRWIPVRYLIISACSCSS-
Cm_Ng1(XP_007909823.1) YTHCPVLYTWKDLGDRFWPRYIREGSCY-SGRSCSFPEGMSCKKAKASTKTLLRWHCPRREL-----HRPCAWIPVQYSIISECKCSC-
Gg_Ng2(AAX07476.1) YTYCPVLYTWKDLGVRFWPRYIKEGNCF-AEKSCSLPEGMFCKPVKSVTKTFLRWHCQGWSS-----QKYCTWIPVQYPLISECKCSC-
Xl_Ng2(AAX07470.1) YTYCPVMYTWKDLGVRFWPRFIKEGHCF-SEKSCSFPEGMYCKPIKSVTKTFLRWYCQGWTR-----QKYCTWIPVQYPIISECKCSC-
Sc_Ng2(XP_038676778.1) YTYCPVMYTWKDLGGRFWPRFVKEGNCY-NERSCSFPEGMLCKPVKSISKTFLRWYCQGWSK-----PKYCTWIPVQYPVISECKCSC-
Rt_Ng2(XP_020376495.2) YTYCPVMYTWKDLGGRFWPRFVKEGNCY-NERSCSFPEGMLCKPVKSISKTFLRWYCQGWSK-----PKYCTWIPVQYPVISECKCSC-
Cp_Ng2(XM_043711557.1) YTYCPVMYTWKDLGGRFWPRFVKEGNCY-NERSCSFPEGMLCKPVKSISKTFLRWYCQGWSK-----PKYCTWIPVQYPVISECKCSC-
Ar_Ng2(XP_032896618.1) YTYCPVVYTWKDLGGRFWPRFVKEGNCY-NERSCSFPEGMLCKPVKSISKTFLRWYCQGWSK-----PRYCTWIPVQYPVISECKCTC-
Cm_Ng2(XP_007891794.1) YTYCPVMYTWKDLGGRFWPRFVKEGNCY-NERSCSFPEGMVCKPVKSINKTFLRWYCQGWSR-----QKYCTWIPVQYPVISECKCSC-
Cc_Ng2(XP_041063000.1) YTYCPVMYTWKDLGGRFWPRFVKEGNCY-NERSCSFPEGMLCKPVKSISKTFLRWYCQGWSK-----PKYCTWIPVQYPVISECKCSC-
Gg_Ng4(AAX07477.1) RASCRLTSAWVDLGPVFWPRWVRHTACRTGPPACSWPPGMACRPAQLAHLKLLAWHCWAARP---PGPPHCAWRQVPYPVVVACKCSCR
Xl_Ng4(AAX07472.1) RASCHLTSSWVDLGTVFWPRWVRHTDCDGANTVCSWPPGMACRQAQLTQIKLLAWHCWMKDTGLGWATQQCTWRQVPYPVVAACKCTCK
Sc_Ng4(XP_038639387.1) VASCPLTSWWVDLGAVFWPRWVRHTDCDGSKVGCSWPPGMTCAQAQWVQIKLLVWHCWAAGE-RARSLRHCTWRQIPYPVVTACKCSCQ
Rt_Ng4(XP_048473669.1) EASCPLTSWWVDLGAVFWPRWVRHTDCDRGRASCSWPPGMSCSQAEWMHIKLLVWHCWTVKE-RVRASQQCTWRQVPYPVVTACKCSCR
Cp_Ng4(XM_043679916.1) EAVCPLTSQWVDLGAIFWPRWVRHTDCDRGRASCFWPPGMSCTQAEWAHIKLLVWHCWTVKE-RGRVSQQCTWRQVPYPVVTACKCSCR
Cm_Ng4(XP_007907783.1) VASCPLTSAWKDLGPVFWPRWVRHTDCDRAKRGCSWPMGMTCQRDRWTHINLLVWHCLAA---RPAVGKHCTWRQIPYPVVTGCKCSCQ**

***Supplementary Figure 1S.*** Noggin protein alignment.

Hs - *Homo sapiens*, Gg - *Gallus gallus*, Xl - *Xenopus laevis*, Cc - *Carcharodon carcharias*, Cm - *Callorhinchus milii*, Sc - *Scyliorhinus canicula*, Rt- *Rhincodon typus*, Cp - *Chiloscyllium plagiosum*

........10........20........30........40........50........60........70........80....
**Hs_Ng(AAA83259.1) 1 ------MERCPSLGVTLYALVVVLGLRATPAGGQHYLHIRPAPSDNLPLVDLIEHPDPIFDPKEKDLNETLLRSLLGGHYDPGF
Gg_Ng1(NP_989454.1) 1 ------MDHSQCL-VTIYAAAVLLGLRLQQGSCQHYLHIRPAPSDNLPLVDLIEHPDPIFDPKEKDLNETLLRSLMGGHFDPNF
Xl_Ng1(AAI69672.1) 1 ------MDHSQCL-VTIYALMVFLGLRIDQGGCQHYLHIRPAPSENLPLVDLIEHPDPIYDPKEKDLNETLLRTLMVGHFDPNF
Lc_Ng1(XP_006000006.1) 1 ------MDHSQCF-LAVYALIVLLGLRIEEGVCQHYLHLRPVPSENLPLVDLIEHPDPVFDPKERDLNDTMLRTILGSNFDPNF
Dr_Ng1(AAD43132.1) 1 ------MDFPRFL-LSAYLLLLSF------AQCQHYYLLRPIPSDTLPLLELKEDPDPIYDPREKDLNETELRSALG-DFDSRF
Lo_Ng1(XP_015211513.1) 1 ------MHHSQYF-IAMYVLVLSLGLTIEEGMCQHYYHLRPIPSDNLPIVELIEHPDPVFDPKERDLNETELRSILGSNFDSHY
Pm_NgA(AEFG01046720.1) 1 ------MELPRHLLTFCTLLAVLMGSQKHRGHCRNYLHFRPSPSDNLPIKDLIENPDPELDPKEQDLDEKLLRRKLGASFDPEF
Eb_NgA (Eptbu0014490.t1) 1 MARAWGAWLPPQVMLPLLVLLWLLLLRVRPARTQHYLHLKPVPSDKLPVADIIEDPDPALDPSEKDTDERVLRRKLGSHFDPDF
Bf_Ng(ABG66526.1) 1 --------MDAWVRLFLLAGSFLICSPAVFG--QPFLHLRPRPSDDLPVLDLIEPPNSEFDPKEADLDVQILRKLLGRQYDPYY
Cm_Ng1(XP_007909823.1) 1 ------MDLSRRR-LTVYLATLLLCVWVRLGAGQHYLHLRPSPSDHLPLLELLEPPDPDLDPKDKDMDETTLRKKLAANFDPNF
Cc_Ng1*(XP_041072794.1) 1 ------------M-LTLYLG-----LWSRSVSGQHYLQLRPAATQHPPLLELWERPDPELDPKDSDLDEVTLRLKLARDLDPDF
Io_Ng1*(JANJGN010000022.1) 1 ------------M-LTLYLG-----LWSRSVSGQHYLQLRPAATQHPPLLELWERPDPELDPKDSDLDEVTLRLKLARDLDPDF
Sa_Ng1”(JASTWF010000530.1) 1 -----MMDRSPYL-LPLYLG-----LWGQLVFGQHYLLLRPSPSDHLPLLELLELPDPELDPKESDLDEATLRQKLASDFDPEY**

....90.......100.......110.......120.......130.......140.......150.......160....... **Hs_Ng(AAA83259.1) 79 MATSPPEDRPGGGGGAAGGAEDLAELDQLLRQRPSGAMPSEIKGLEFSEGLAQGKKQRLSKKLRRKLQMWLWSQTFCPVLYAWN
Gg_Ng1(NP_989454.1) 78 MAMSLPEDRLG--------VDDLAELDLLLRQRPSGAMPGEIKGLEFYDGLQPGKKHRLSKKLRRKLQMWLWSQTFCPVLYTWN
Xl_Ng1(AAI69672.1) 78 MATILPEERLG--------VEDLGELDLLLRQKPSGAMPAEIKGLEFYEGLQS-KKHRLSKKLRRKLQMWLWSQTFCPVLYTWN
Lc_Ng1(XP_006000006.1) 78 MSFSLPDERLAG-------NEDLAELDLTLRQKPTGVMPSEIKLLEFYDALHG-KKHRLSKKLRRKLQMWLWSHTFCPVLYTWD
Dr_Ng1(AAD43132.1) 71 LSVGPPQDRYAG-------NEDLDEQEL--QLNLAGMMPKDIKNLDF-DAPWG-KKRKASKKLKRRLQMWLWSYSFCPVLYAWN
Lo_Ng1(XP_015211513.1) 78 MSVSPPEDKYTG-------NDELADLEL--RQKPTGAMPKEIKSMEFFDFLHG-KKHKPSKKLRRRLQLWLWSYAFCPVVYTWN
Pm_NgA(AEFG01046720.1) 79 MAVSLP-----------KGDASGQQTRGGRLLKPSGSMPNEIKRLDLGVLPHG-QKIKIGKRARRKILQWLWSYTFCPVLYTWK
Eb_NgA (Eptbu0014490.t1) 85 MAVSRPPLIVDP----SPTSVSTSWRSKPRFQRPLGAVPPELRRLDLSETALG-QQLKLGKRVRRKLLQWLWARTACPVRYTWK
Bf_Ng(ABG66526.1) 75 MSIEHPDPAMLH----ANCTLNGESNEGNRCK-PPGPKPRWIDDLDVGTLPSG-RKVKLSRKSRRKFKMWMWSLTHCPVVHSWK
Cm_Ng1(XP_007909823.1) 78 MALRLPEGWEP----------EAGAPEPGLRHRPGGFMPNHIRRVDFVVAAGR--KQRLSKKLRRRLQLWLWSYTHCPVLYTWK
Cc_Ng1*(XP_041072794.1) 67 MSPRFP-GPEP----------VPR------------------------------------------------------------
Io_Ng1*(JANJGN010000022.1) 67 MSPWFL-GPEL----------VPRVSEG---------VPDTAGRDEPGSAP-----HWVSRKGWRWFELRLQDRSRCPVT*SWR
Sa_Ng1”(JASTWF010000530.1) 74 MSLRPPEGGRT----------VPDPGP----------IPGHIRRIELGPQ------QRLKGKGRRRLQQWLWGRTRCPVSYRWR**

170.......180.......190.......200.......210.......220.......230.......240.......250..
**Hs_Ng(AAA83259.1) 163 DLGSRFWPRYVKVGSCFSKRSCSVPEGMVCKPSKSVHLTVLRWRC-QRRGG----------QRCGWIPIQYPIISECKCSC---
Gg_Ng1(NP_989454.1) 154 DLGSRFWPRYVKVGSCYSKRSCSVPEGMVCKPAKSVHLTILRWRC-QRRGG----------QRCTWIPIQYPIIAECKCSC---
Xl_Ng1(AAI69672.1) 153 DLGTRFWPRYVKVGSCYSKRSCSVPEGMVCKAAKSMHLTILRWRC-QRRVQ----------QKCAWITIQYPVISECKCSC---
Lc_Ng1(XP_006000006.1) 154 DLGTRFWPRYVKVGSCYTKRSCSVPEGMVCKPAKSVHLTILRWRC-QRRGG----------QKCTWIPVQYPIISECKCSCQNF
Dr_Ng1(AAD43132.1) 144 DLGSRFWPRFVRAGSCYTKRSCSVPEGMVCKPAKSTHITLLRWRCVARRGA----------LKCAWIPVQYPIITECKCSCAN-
Lo_Ng1(XP_015211513.1) 152 DLGNRFWPRYVKVGSCYSKRSCSVPEGMVCKPAKSAHFTILRWRCLQRKTG----------QKCAWIPIQYPIISECKCSCSN-
Pm_NgA(AEFG01046720.1) 151 DLGERFWPRFVKEGSCYNGRSCSFPEGMACKPFKSASKTLLRWHCQGWGRQ----------KYCAWIHIQYPVISECRCAC---
Eb_NgA (Eptbu0014490.t1) 164 ELGARFWPRFVKEGTCSRARSCSVPAGMVCRPSGSVSKTLLRWHCQGWERQ----------RFCTWITFQYPVISECKCAC---
Bf_Ng(ABG66526.1) 153 DLGVRFWPRWVKEGRCSTGRSCSFPPGMTCRPSRSVMKTFLRFHCQGWGKQ----------HNCNWIKIHYPLIAECSCSC---
Cm_Ng1(XP_007909823.1) 150 DLGDRFWPRYIREGSCYSGRSCSFPEGMSCKKAKASTKTLLRWHCPRRELH----------RPCAWIPVQYSIISECKCSC---
Cc_Ng1*(XP_041072794.1) 80 -----------------------------------------------WDRE----------RHCRWIPVRYLIISACSCSS---
Io_Ng1*(JANJGN010000022.1) 125 DLG----TRFIREGLGSRVHSCSIPVGLTYKQNKCISKTQLSWNCPQWDRE----------RHCRWIQVRYLIISACSCSS---
Sa_Ng1”(JASTWF010000530.1) 132 DLGRRFWPRFIKEGRCSRRRSCSFPQGMTCKKAKSASKTVLRWHCPHRDREPHRDREPHRDRDCRWVPVQYSIISGCSCSC---**

***Supplementary Figure 2S.*** Noggin1 proteins alignment.

Hs - *Homo sapiens*, Gg - *Gallus gallus*, Xl - *Xenopus laevis*, Lc – *Latimeria chalumnae*, Dr – *Danio rerio*, Lo – *Lepisosteus oculatus*, Eb –*Eptatretus burgeri*, Pm – *Petromyzon marinus*, Bf – *Branchiostoma floridae*, Io – *Isurus oxyrinchus* Sa – *Squalus acanthias*, Cc - *Carcharodon carcharias*, Cm - *Callorhinchus milii*, Sc - *Scyliorhinus canicula*, Rt- *Rhincodon typus*, Cp - *Chiloscyllium plagiosum*


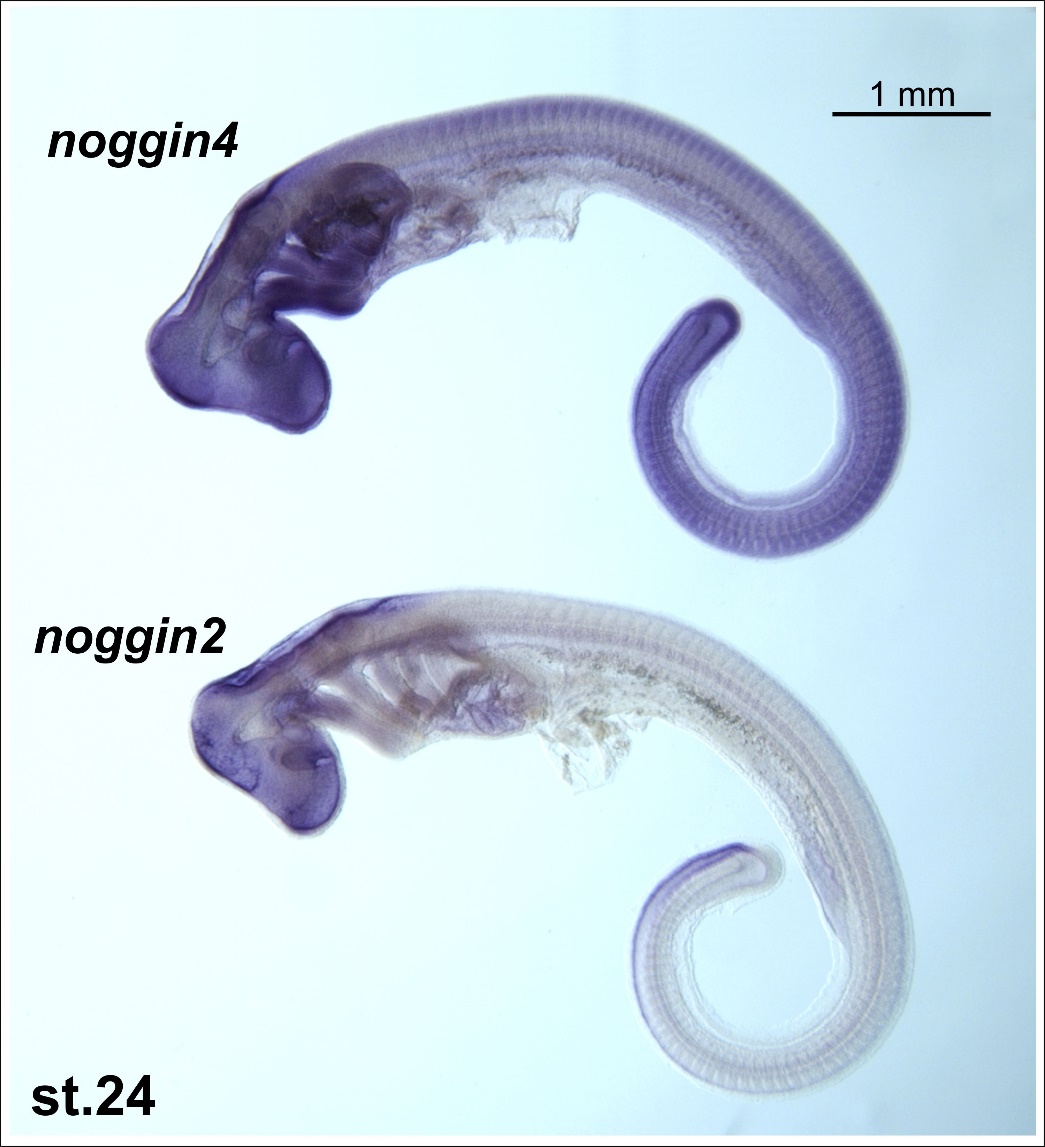


***Supplementary Figure 3S***. Difference of *noggin4* and *noggin2* expression in somites of *C. griseum* embryos at stage 24.


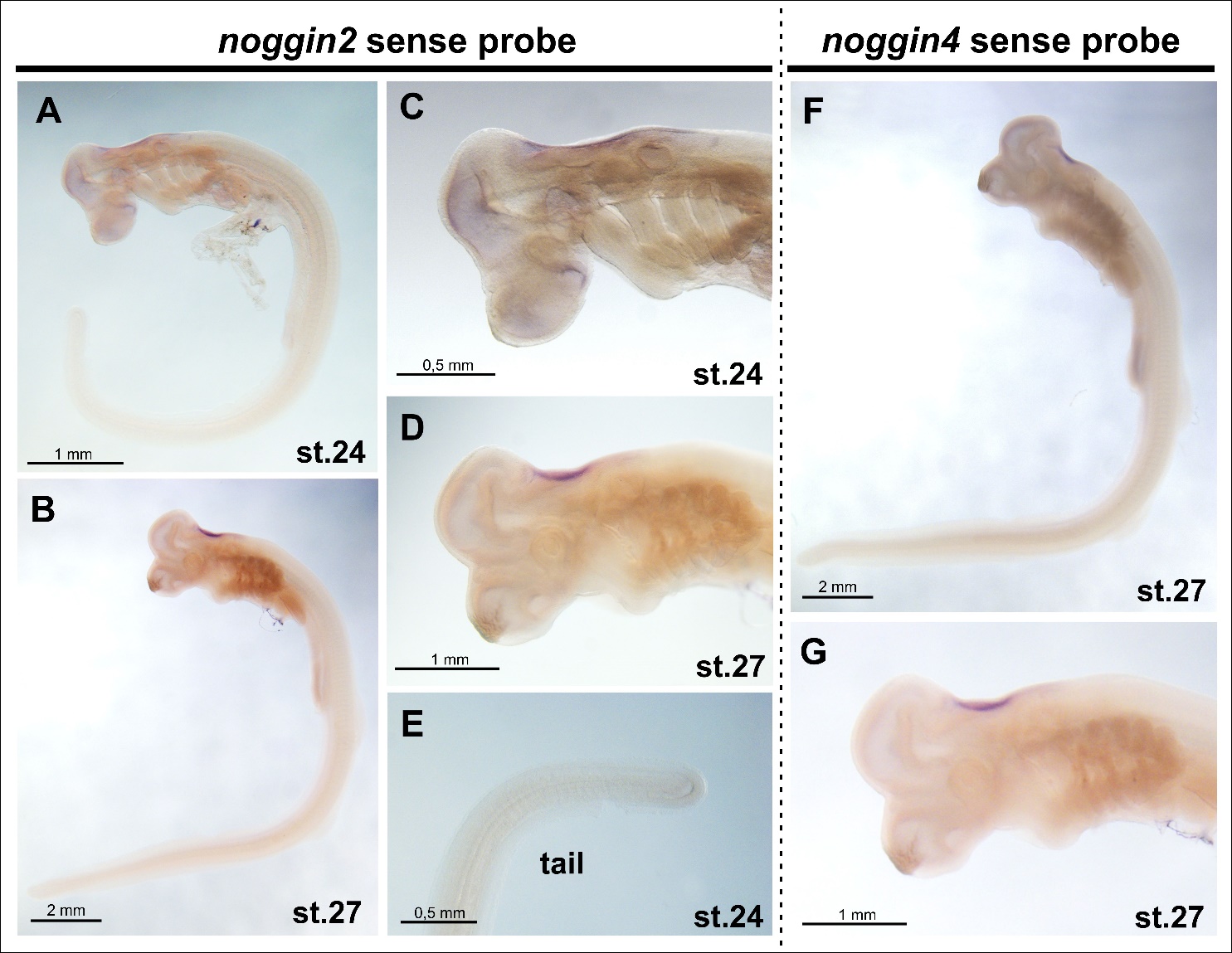


***Supplementary Figure 4S***. ISH performed with sense probes of *noggin4* and *noggin2*.


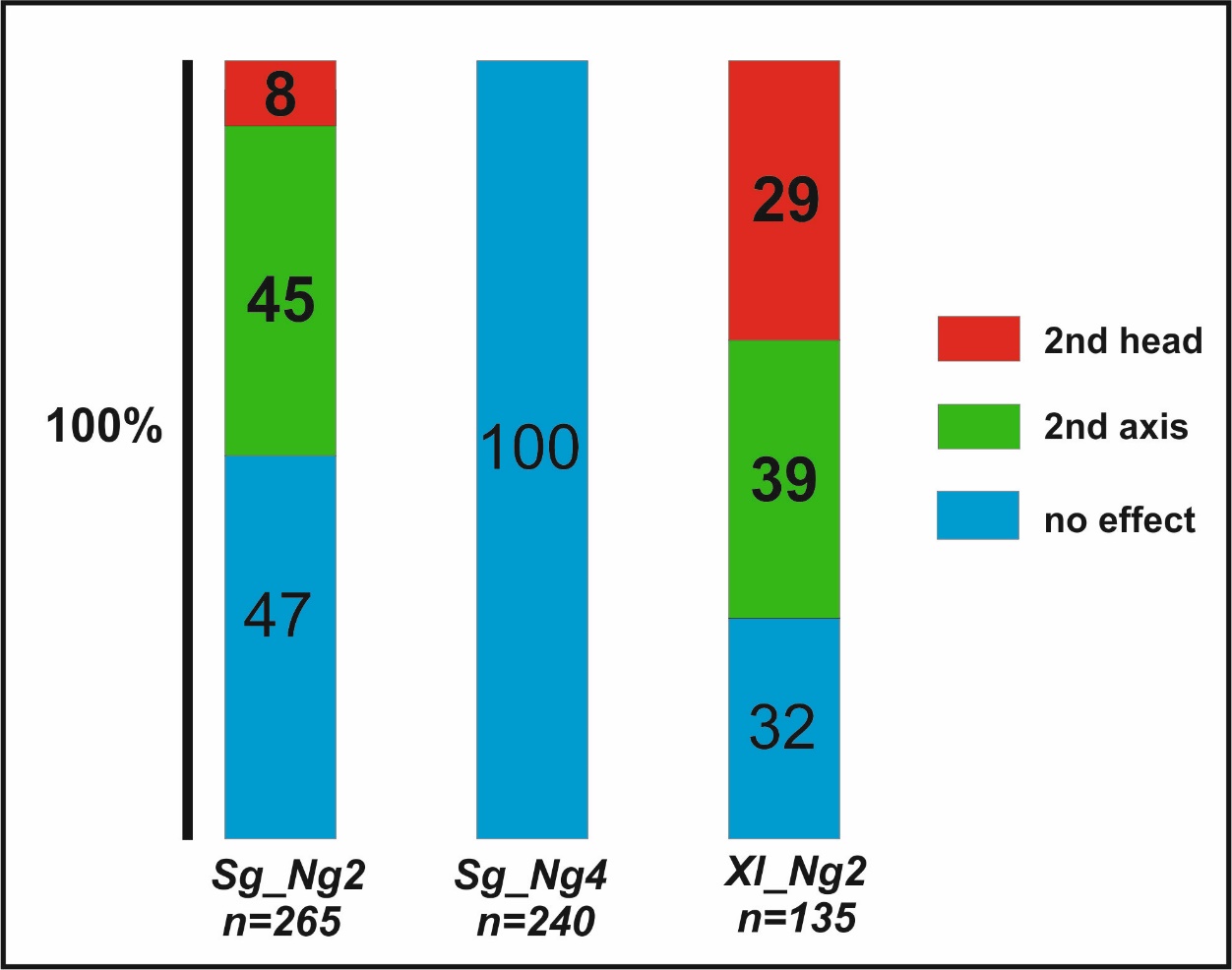


***Supplementary Figure 5S.*** Percentage of secondary axes induction by *S. griseum noggin2* and *noggin4* mRNAs injections in *X. laevis* embryos in comparison with *X. laevis noggin2* mRNA.

**Supplementary File 1.** Chondrichthyes Noggin1

> *C. milii* Noggin1 (XP_007909823.1)

MDLSRRRLTVYLATLLLCVWVRLGAGQHYLHLRPSPSDHLPLLELLEPPDPDLDPKDKDMDETTLRKKLAANFDPNFMALRLPEGWEPEAGAPEPGLRHRPGGFMPNHIRRVDFVVAAGRKQRLSKKLRRRLQLWLWSYTHCPVLYTWKDLGDRFWPRYIREGSCYSGRSCSFPEGMSCKKAKASTKTLLRWHCPRRELHRPCAWIPVQYSIISECKCSC

> *S. acanthias* Noggin1 (JASTWF010000530.1)

MMDRSPYLLPLYLGLWGQLVFGQHYLLLRPSPSDHLPLLELLELPDPELDPKESDLDEATLRQKLASDFDPEYMSLRPPEGGRTVPDPGPIPGHIRRIELGPQQRLKGKGRRRLQQWLWGRTRCPVSYRWRDLGRRFWPRFIKEGRCSRRRSCSFPQGMTCKKAKSASKTVLRWHCPHRDREPHRDREPHRDRDCRWVPVQYSIISGCSCSC

>*C. carcharias* Noggin1 translated pseudogene (JAGDEE010000072.1)

MLTLYLGLWSRSVSGQHYLQLRPAATQHPPLLELWERPDPELDPKDSDLDEVTLRLKLARDLDPDFMSPRFPGPEPVPRVSEGAPYTAGWDEPRSAPHWVSRKGWRWFELWLQDRSRYPVT*SWRDLGTRFIREGPGSRVHSCSIPVGLTCKQNKCISKTQLS*YCPQWDRERHCRWIPVRYLIISACSCSS

>*I. oxyrinchus* Nggin1 translated pseudogene (JANJGN010000022.1)

MLTLYLGLWSRSVSGQHYLQLRPAATQHPPLLELWERPDPELDPKDSDLDEVTLRLKLARDLDPDFMSPWFLGPELVPRVSEGVPDTAGRDEPGSAPHWVSRKGWRWFELRLQDRSRCPVT*SWRDLGTRFIREGLGSRVHSCSIPVGLTYKQNKCISKTQLSWNCPQWDRERHCRWIQVRYLIISACSCSS

**Supplementary Table 1** – Results of the models testing for phylogenetic analysis.
